# Photon-counting detector computed tomography for metal artifact reduction: a comparative study of different artifact reduction techniques in patients with orthopedic implants

**DOI:** 10.1007/s11547-024-01822-x

**Published:** 2024-04-30

**Authors:** Fabian Bernhard Pallasch, Alexander Rau, Marco Reisert, Stephan Rau, Thierno Diallo, Thomas Stein, Sebastian Faby, Fabian Bamberg, Jakob Weiss

**Affiliations:** 1 Department of Radiology, University Medical Center Freiburg, Hugstetter Str. 55, 79106 Freiburg im Breisgau, Germany; 2 Siemens Healthcare GmbH, Siemensstr. 3, 91301 Forchheim, Germany

**Keywords:** Multidetector computed tomography, Artifacts, Image processing, Computer-assisted, Arthroplasty, replacement, hip, Spine

## Abstract

**Purpose:**

Artifacts caused by metallic implants remain a challenge in computed tomography (CT). We investigated the impact of photon-counting detector computed tomography (PCD-CT) for artifact reduction in patients with orthopedic implants with respect to image quality and diagnostic confidence using different artifact reduction approaches.

**Material and methods:**

In this prospective study, consecutive patients with orthopedic implants underwent PCD-CT imaging of the implant area. Four series were reconstructed for each patient (clinical standard reconstruction [PCD-CT_Std_], monoenergetic images at 140 keV [PCD-CT_140keV_], iterative metal artifact reduction (iMAR) corrected [PCD-CT_iMAR_], combination of iMAR and 140 keV monoenergetic [PCD-CT_140keV+iMAR_]). Subsequently, three radiologists evaluated the reconstructions in a random and blinded manner for image quality, artifact severity, anatomy delineation (adjacent and distant), and diagnostic confidence using a 5-point Likert scale (5 = excellent). In addition, the coefficient of variation [CV] and the relative quantitative artifact reduction potential were obtained as objective measures.

**Results:**

We enrolled 39 patients with a mean age of 67.3 ± 13.2 years (51%; n = 20 male) and a mean BMI of 26.1 ± 4 kg/m^2^. All image quality measures and diagnostic confidence were significantly higher for the iMAR vs. non-iMAR reconstructions (all *p* < 0.001). No significant effect of the different artifact reduction approaches on CV was observed (*p* = 0.26). The quantitative analysis indicated the most effective artifact reduction for the iMAR reconstructions, which was higher than PCD-CT_140keV_ (*p* < 0.001).

**Conclusion:**

PCD-CT allows for effective metal artifact reduction in patients with orthopedic implants, resulting in superior image quality and diagnostic confidence with the potential to improve patient management and clinical decision making.

**Supplementary Information:**

The online version contains supplementary material available at 10.1007/s11547-024-01822-x.

## Introduction

Artifacts caused by metallic orthopedic implants such as prosthesis and osteosynthesis are an ongoing challenge in computed tomography (CT) imaging, and their prevalence is expected to further increase in an aging population [1, 2]. These artifacts manifest as streaks and shadows around the implant due to photon starvation, photon scattering, and beam hardening [3, 4]. As a result, image quality can be substantially impaired with the risk of obscuring important findings like implant failure, fractures, or malignancies [5, 6]. Consequently, various approaches were developed and studied to overcome these challenges and to effectively reduce artifacts. These approaches include the calculation of virtual monoenergetic reconstructions at high keV, sinogram inpainting, and iterative metal artifact reduction (iMAR) approaches [7–11]. Alone or in combination, these strategies have demonstrated encouraging results in different patient populations (e.g., patients with dental implants, pacemakers, cerebral aneurysm clips, and orthopedic implants) and are increasingly used in daily routine to improve clinical decision making [7, 11–15].

With the clinical implementation of photon-counting-detector CT (PCD-CT) in late 2021, a new technology with the potential to fundamentally change CT imaging has become available [16]. Conventional energy-integrating detectors (EID) rely on scintillators to convert photon energy into electric current for image reconstruction. In contrast, photon-counting detectors directly convert the detected signal and allow for counting and measuring individual photons and their respective energy level [17]. As a result, spectral data is intrinsically obtained in each scan with the potential to improve image post-processing compared to the established EID-CT technology [16, 17]. PCD-CT with multiple energy bins present an advantage compared to EID-CT since different energy bins capture distinct attenuation properties. In the context of metal artifact reduction, high energy-bin images exhibit fewer beam artifacts compared to low-energy-bin images and EID-CT [18]. Another advantage is that reflective septa are not required in PCD setup with the potential to reduce radiation dose compared to EID-CT systems [17, 19]. Finally, dual-energy EID-CT systems always require an upfront decision whether to obtain images in dual- or single-energy mode with consequences on available post-processing possibilities. In contrast, PCD-CT data always allows for advanced post-processing in every scan (e.g., high keV reconstructions), which can be useful for further clarification, especially if unexpected findings are detected. This is supported by a constantly growing body of research demonstrating superior image quality for PCD-CT in different clinical applications [20–26].

Therefore, this study aimed to investigate and compare image quality and diagnostic confidence of PCD-CT for metal artifact reduction using different approaches (monoenergetic reconstructions at 140 keV and iMAR algorithm as standalone techniques and combined) in patients with orthopedic implants. We hypothesized that PCD-CT can effectively reduce artifacts allowing for a significantly improved image quality and diagnostic confidence.

## Material and methods

### Patient population

The local ethics committee approved this retrospective analysis of prospectively acquired data and written informed consent was obtained from all participants. Consecutive oncological patients with orthopedic implants and indication for PCD-CT imaging as part of their routine clinical workup were enrolled in this study between July 11th, 2022 and August 31st, 2022. Patients were excluded if they had contraindications for contrast-enhanced CT imaging such as allergy to iodine contrast agents, renal impairment, and thyroid dysfunction, as well as individuals under 18 years of age.

### Imaging protocol and reconstruction parameters

All CT scans were performed in a supine position using a first-generation dual-source PCD-CT system (NAEOTOM Alpha, Siemens Healthcare, Forchheim, Germany). For all patients, portal-venous phase images were acquired 75 s after body weight-adapted contrast agent administration (Ultravist 370 Bayer Healthcare, 1,2 mg/kg; flow 2 ml/s) using a dual-syringe power injector (Medtron, Saarbruecken, Germany) followed by a 20 ml saline bolus (flow 2 ml/s).

CT data were acquired in multi-spectral mode with a pitch of 0.8, a gantry rotation time of 0.25 s, a collimation of 144 × 0.40 mm, and a CARE keV IQ level = 145 (corresponding to a reference mAS ranging from 55 to 100). In addition, CareDose4D and CARE keV were enabled for all examinations.

For further analyses, four axial series were reconstructed from the acquired multi-spectral data:

(i) Standard reconstruction (PCD-CT_Std_) at a monoenergetic level of 65 keV to simulate a conventional EID-CT image impression of 120 keV with a bone kernel (Br56), a slice thickness and increment of 2 mm, and quantum iterative reconstruction strength 4.
(ii) High keV monoenergetic reconstruction at 140 keV (PCD-CT_140keV_) following previously published results by Anhaus et al. [8], other parameters as in (i).
(iii) IMAR reconstruction (PCD-CT_iMAR_) using a commercially available iMAR algorithm (Siemens Healthineers, Erlangen, Germany), other parameters as in (i). The iMAR algorithm was specifically developed for efficient metal artifact reduction by combining several recently proposed techniques in an iterative process. In detail, beam hardening caused by the implant is addressed through correction and inpainting in raw data space using a normalized sinogram. In addition, a frequency split technique is applied in the image domain for further and robust artifact reduction [10, 27]. There are seven dedicated optimized iMAR algorithms available for hip implants, intracranial coils, thoracic coils, shoulder implants, pacemakers, dental fillings, and extremity implants. For the current study, the hip implant algorithm was used for all patients as no dedicated application for spinal implants is currently available.
(iv) A combined reconstruction applying the iMAR algorithm to monoenergetic reconstructions at 140 keV (PCD-CT_140keV+iMAR_), other parameters as in (i).

### Qualitative image analysis

The image quality of all the reconstructions was subjectively evaluated by three radiologists with 2, 3, and 5 years of experience in CT imaging. All ratings were performed on a dedicated workstation equipped with in-house research image processing software (Nora; https://www.nora-imaging.com/). To ensure unbiased reading results, the radiologists independently assessed all reconstructions on 5-point Likert scales, which were presented in a random order and blinded regarding the type of reconstruction.

**Fig. 1:**
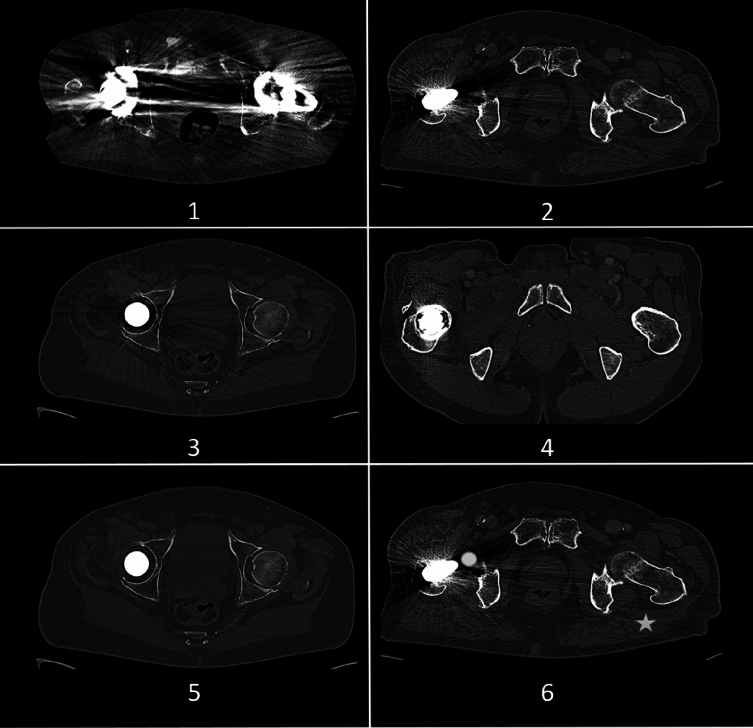
Example of subjective image quality analysis using a 5-Point Likert scale for overall image quality, artifact severity, delineation of adjacent as well as distant anatomy, and diagnostic confidence, which were graded as: Panel 1—non-diagnostic, Panel 2—poor/severe artifacts, Panel 3—fair/moderate artifacts, Panel 4—good/minor artifacts, Panel 5—excellent/no artifacts. Panel 6 displays the ROI measurements for the coefficient of variation (star) and quantitative artifact reduction analysis (circle—placed in the artifact core)

The following criteria were evaluated (i) overall image quality; (ii) artifact severity; (iii) delineation of adjacent anatomy (vessels, bone, musculature, lymph nodes); and (iv) delineation of distant anatomy (distant muscles, abdominal/pelvic organs, vessels); (v) diagnostic confidence and interpreted as 5 = excellent/no artifacts, 4 = good/minor artifacts, 3 = fair/moderate artifacts, 2 = poor/severe artifacts, 1 = non-diagnostic (see Fig. 1).

Results are reported for the entire cohort and stratified by hip implants versus spinal osteosyntheses to investigate whether the hip implant iMAR algorithm could be employed as a reliable alternative for the currently lacking dedicated approach for spinal osteosyntheses.

### Quantitative analysis

#### Coefficient of variation (CV)

The CV was calculated to obtain quantitative image measures and examine possible alterations in image signal homogeneity by the different artifact techniques. CV, also known as the relative standard deviation, is a statistical measure used to assess the relative variability of a dataset in relation to its mean. Therefore, a region-of-interest (ROI) 150 mm^2^ in size was placed in a distant muscle, which was subjectively least by the artifact. The localization was identical across all reconstructions (PCD-CT_Std_, PCD-CT_140keV_, PCD-CT_iMAR_, PCD-CT_140keV+ iMAR_) and all measurements were performed in a random order and blinded to the type of reconstruction to ensure a reliable comparison. Mean Hounsfield units (HU) and standard deviations (SD) were measured from the ROI and the CV was calculated using the following equation:

1 $$ {\text{CV}}[{\text{HU}}]{ } = {\text{ SD}}_{{\text{ROI}}} { }/{\text{ HU}}_{{\text{ROI}}} { } $$

where CV = the coefficient of variation, SD = standard deviation of the ROI, and HU = the mean density in of the ROI.

#### Quantification of artifact reduction

To quantitatively assess the effectiveness of the different methods for artifact reduction, we calculated the relative change in artifact severity based on changes in HU within the artifact using the PCD-CT_Std_ reconstruction as a reference. Firstly, the image with the most severe artifact was selected (by XX) on the PCD-CT_Std_ reconstruction for all patients. Secondly, the corresponding images in PCD-CT_140keV_, PCD-CT_iMAR_, and PCD-CT_140keV+iMAR_ reconstructions were defined. Finally, a circular ROI (150 mm^2^) was used to measure the mean HU values within the artifact in all reconstructions (Fig. 1). The relative change in HU values as a ratio between the different artifact reduction techniques was then calculated as follows:

2 $$ Ratio{ } = { }(PCD - CT_{reduction\_technique} { }/{ }PCD - CT_{Std} ){ * }100{ } $$

where ratio = the relative potential for artifact reduction, PCD-CT_reduction_technique_ = one of the investigated artifact reduction techniques, and PCD-CT_std_ = the standard reconstruction without artifact reduction.

Similar to the qualitative analysis, we conducted a subgroup analysis stratifying hip implants and spinal osteosyntheses.

### Statistical analysis

All statistical analyses were performed using the R Foundation for Statistical Computing (version 4.2.1, Vienna, Austria). Continuous variables are given as mean and SD or median and interquartile ranges, as appropriate. Categorical measures are presented as percentages and frequencies. Friedman's ANOVA with post hoc pairwise comparison was calculated to compare the results of the qualitative image analysis for the entire cohort and stratified by patients with hip implants vs. spinal osteosyntheses. For quantitative measures, repeated measure ANOVA followed by post hoc pairwise comparison was conducted. For all comparisons, Bonferroni correction was applied to account for multiple testing. P values were considered to indicate statistical significance if < 0.05.

## Results

The final study cohort consisted of 39 consecutive patients with a mean age of 67.2 ± 13.2 years, 51% male, and a mean BMI of 26.1 ± 4 kg/m^2^. For all patients, the four types of reconstructions were successfully calculated. No patients were excluded. The mean CTDI_vol_ was 8 ± 4.3 mGy. Detailed patient characteristics are provided in Table 1.

**Table 1:** Patient characteristics and radiation dose

Variable	N (%) or mean ± SD
Participants	39 (100%)
Sex (male)	20 (51%)
Age (years)	67.2 ± 13.2
Weight (kg)	74.3 ± 14
Height (meter)	1.69 ± 0.1
BMI (kg/m^2^)	26.1 ± 4
Metallic implants	
Spinal osteosynthesis	13 (33.3%)
Hip replacement	13 (33.3%)
Osteosynthesis	9 (23%)
Knee replacement	2 (5.1%)
Shoulder replacement	2 (5.1%)
Clinical diagnosis	
GI-cancer	8 (20.5%)
Orthopedic	8 (20.5%)
Breast cancer	6 (15.4%)
Vascular	6 (15.4%)
Lung cancer	6 (15.4%)
Hematologic neoplasia	5 (12.8%)
Radiation dose	
CTDIvol (mGy)	8 ± 4.3

### Qualitative analysis

All radiologists completed their reading sessions for all patients and reconstructions. An overview of the results is presented in Table 2.

**Table 2:** Results of the qualitative image analysis

	PCD-CT_std_	PCD-CT_140 keV_	PCD-CT_iMAR_	PCD-CT_140keV+iMAR_
Image quality	3 [2.7–3]	3.7 [3.2–3.7]	4 [3.5–4.3]	4.3 [3.8–4.67]
*Comparison*				
PCD-CT_std_	–			
PCD-CT_140 keV_	*p* = 0.25	–		
PCD-CT_iMAR_	*p* = 0.003	*p* = 0.25	–	
PCD-CT_140keV+iMAR_	*p* < 0.001	*p* = 0.051	*p* = 1	–
Artifact severity	2 [1.7–2.3]	3 [2.5–3.7]	3.7 [3–4]	4 [3.3–4.7]
*Comparison*				
PCD-CT_std_	–			
PCD-CT_140 keV_	*p* = 0.25	–		
PCD-CT_iMAR_	*p* < 0.01	*p* = 0.52	–	
PCD-CT_140keV+iMAR_	*p* < 0.01	*p* = 0.25	*p* = 1	–
Adjacent anatomy	2 [1.7–2.3]	3 [2.5–3.7]	3.7 [3–4]	4 [3.3–4.7]
*Comparison*				
PCD-CT_std_	–			
PCD-CT_140 keV_	*p* = 0.92	–		
PCD-CT_iMAR_	*p* < 0.01	*p* = 0.27	–	
PCD-CT_140keV+iMAR_	*p* < 0.001	*p* < 0.01	*p* = 0.43	–
Distant anatomy	2 [1.7–2.3]	3 [2.5–3.7]	3.7 [3–4]	4 [3.3–4.7]
*Comparison*				
PCD-CT_std_	–			
PCD-CT_140 keV_	*p* = 0.02	–		
PCD-CT_iMAR_	*p* = 0.02	*p* = 1	–	
PCD-CT_140keV+iMAR_	*p* < 0.001	*p* = 1	*p* = 0.88	–
Diagnostic confidence	2 [1.7–2.3]	3 [2.5–3.7]	3.7 [3–4]	4 [3.3–4.7]
*Comparison*				
PCD-CT_std_	–			
PCD-CT_140 keV_	*p* = 1	–		
PCD-CT_iMAR_	*p* = 0.07	*p* = 0.566	–	
PCD-CT_140keV+iMAR_	*p* < 0.001	*p* = 0.003	*p* = 0.28	–

PCD-CT_Std_ = Standard reconstruction; PCD-CT_140keV_ = virtual monoenergetic reconstruction at 140 keV; PCD-CT_iMAR_ = dedicated iterative metal artifact reduction algorithm; PCD-CT_140keV+iMAR_ = dedicated iterative metal artifact reduction algorithm combined with virtual monoenergetic reconstruction at 140 keV

Friedman's ANOVA revealed a significant effect of the different reconstruction techniques on image quality for all evaluated criteria. Results were as follows: overall image quality (Friedman’s Q (df = 3) 23.4; *p* < 0.001), artifact severity (Friedman’s Q (df = 3) 26; *p* < 0.001), delineation of adjacent (Friedman’s Q (df = 3) 24.6; *p* < 0.001), delineation of distant anatomy (Friedman’s Q (df = 3) 18.2; *p* < 0.001); diagnostic confidence (Friedman’s Q (df = 3) 21.9; *p* < 0.001). The highest reading scores were found for PCD-CT_140keV+iMAR_ across all evaluated criteria followed by PCD-CT_iMAR_ and the non-iMAR reconstructions. Bonferroni corrected post hoc pairwise testing revealed significantly higher scores for PCD-CT_140keV+iMAR_ vs. PCD-CT_Std_ (all *p* ≤ 0.006) and except for diagnostic confidence (*p* = 0.06) PCD-CT_iMAR_ versus PCD-CT_Std_ were significant (*p* ≤ 0.02). Scores for PCD-CT_140keV_ were significantly higher compared to PCD-CT_Std_ for the assessment of the distant anatomy (*p* = 0.02). A representative example is given in Table 2.

**Fig. 2:**
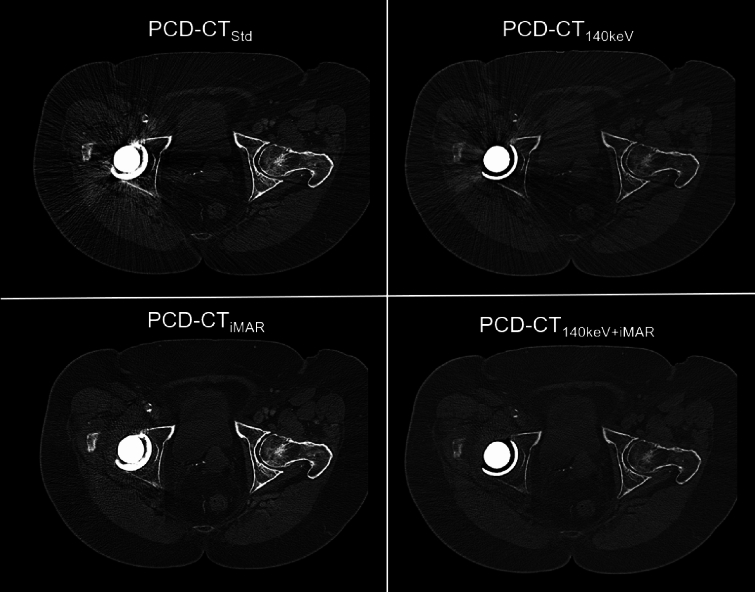
Image example of a 71-year-old patient with non-small cell lung cancer and hip replacement on the right side. While PCD-CT_140keV_ only provided minor artifact reduction PCD-CT_iMAR_ and PCD-CT_iMAR+140 keV_ showed substantially more effective artifact reduction. PCD-CT_Std_ = Standard reconstruction; PCD-CT_140keV_ = virtual monoenergetic reconstruction at 140 keV; PCD-CT_iMAR_ = dedicated iterative metal artifact reduction algorithm; PCD-CT_140keV+iMAR_ = dedicated iterative metal artifact reduction algorithm combined with virtual monoenergetic reconstruction at 140 keV

**Fig. 3:**
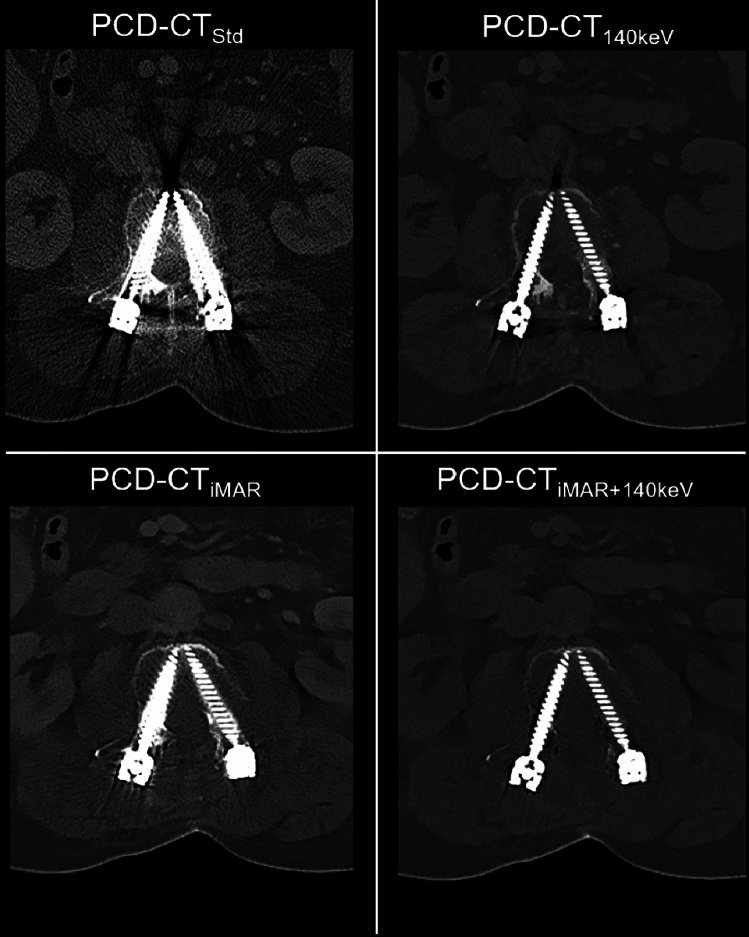
Image example of a 76-year-old patient with breast cancer and spinal osteosynthesis. PCD-CT_140keV_ demonstrated superior artifact reduction for spinal osteosynthesis compared to iMAR alone; most effective artifact reduction was achieved with PCD-CT_140keV + iMAR_ likely because there is currently no dedicated spinal iMAR algorithm available. PCD-CT_Std_ = Standard reconstruction; PCD-CT_140keV_ = virtual monoenergetic reconstruction at 140 keV; PCD-CT_iMAR_ = dedicated iterative metal artifact reduction algorithm; PCD-CT_140keV+iMAR_ = dedicated iterative metal artifact reduction algorithm combined with virtual monoenergetic reconstruction at 140 keV

Subgroup analysis stratified by hip replacements vs. spinal osteosyntheses revealed a similar pattern for patients with hip replacements (Table S1, image example for hip replacement Fig. 2). For spinal osteosynthesis, reading scores for PCD-CT_140keV_ were substantially higher compared to the hip replacement analysis resulting in significant differences vs. PCD-CT_Std_ similar to PCD-CT_iMAR_ and PCD-CT_iMAR+140 keV_, which was not seen for patients with hip replacements (Table S2, image example for spinal osteosynthesis Fig. 3).

### Quantitative image analysis

#### Coefficient of variation

**Fig. 4:**
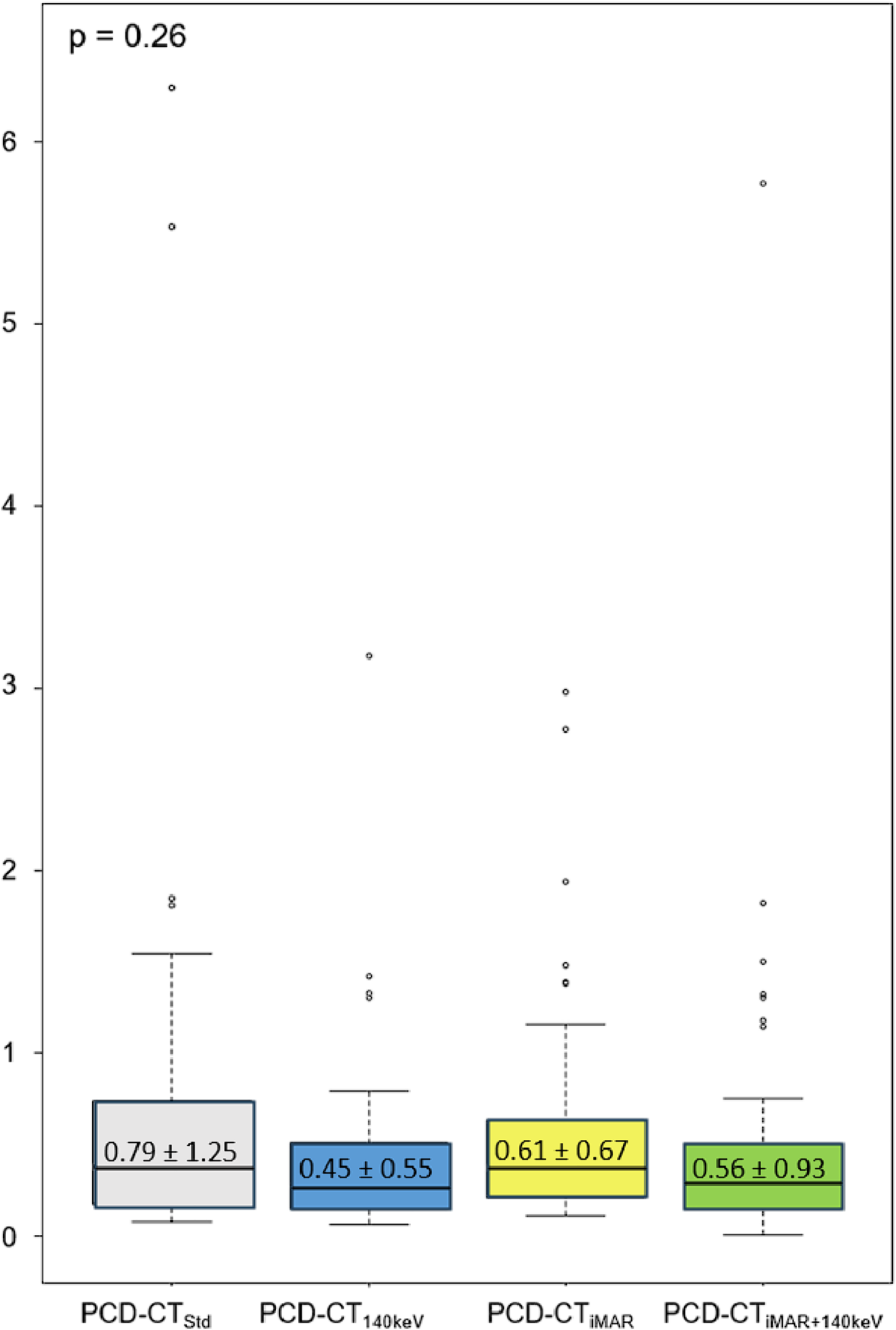
Results of the coefficient of variation analysis as a measure to estimate signal homogeneity. ANOVA revealed no significant impact of the artifact reduction technique on CV (*p* = 0.26). PCD-CT_Std_ = Standard reconstruction (gray); PCD-CT_140keV_ = virtual monoenergetic reconstruction at 140 keV (blue); PCD-CT_iMAR_ = dedicated iterative metal artifact reduction algorithm (yellow); PCD-CT_140keV+iMAR_ = dedicated iterative metal artifact reduction algorithm combined with virtual monoenergetic reconstruction at 140 keV (green)

Mean CV values were 0.79 ± 1.2 for PCD-CT_Std_, 0.45 ± 0.55 for PCD-CT_140keV_, 0.56 ± 0.93 for PCD-CT_iMAR_ = 0.61 ± 0.67, and PCD-CT_140keV+iMAR_. No significant effect for the type of reconstruction on signal homogeneity was noted (repeated measure ANOVA: F value = 1.34; df = 3; *p* = 0.26). An overview is provided in Fig. 4.

#### Quantification of artifact reduction

**Fig. 5:**
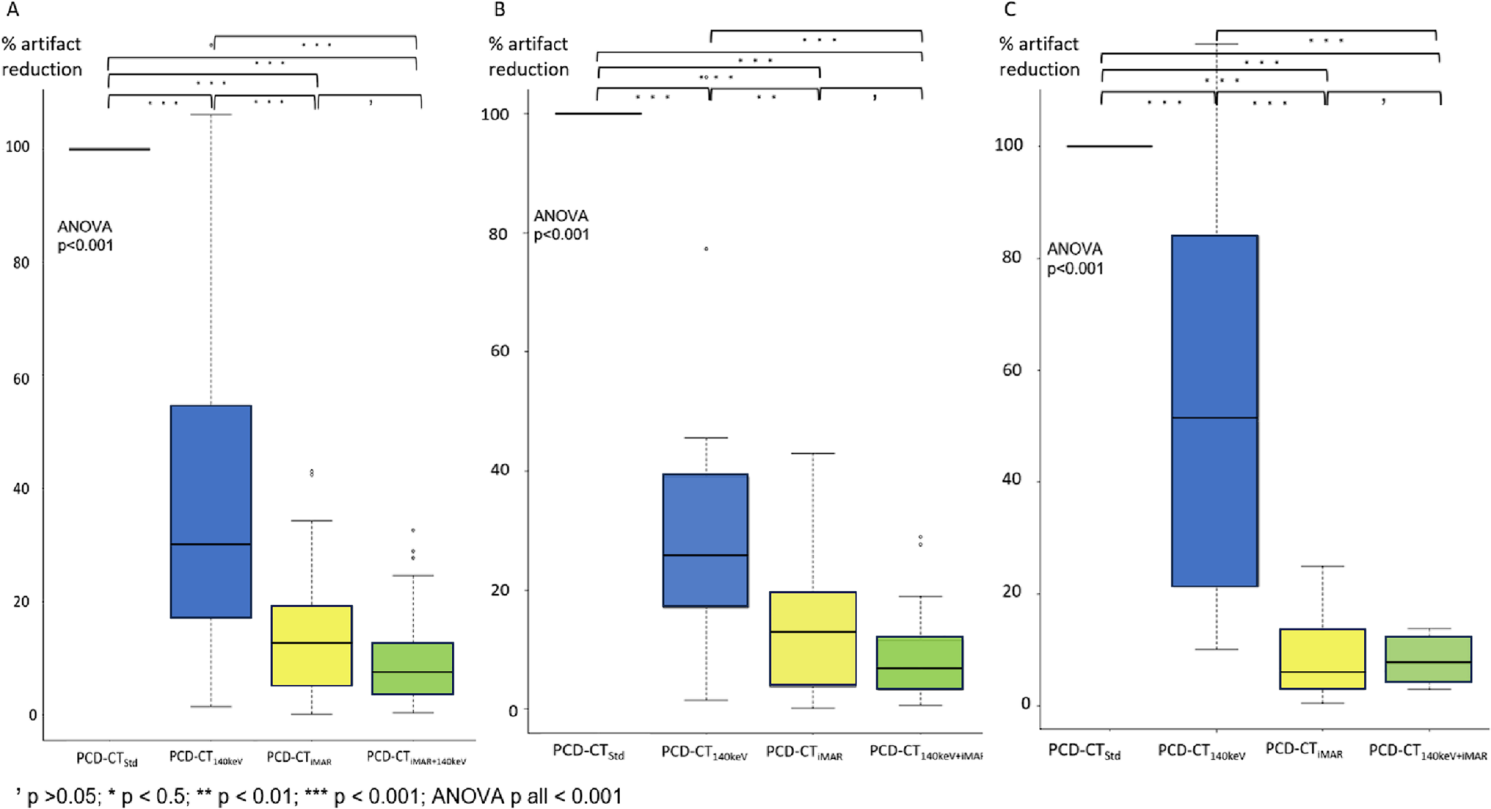
Results of the HU-based approaches to estimate the quantitative artifact reduction potential of the different investigated techniques. Most effective artifact reduction was achieved by PCT-CT_iMAR+140 keV_ followed by PCD-CT_iMAR_ and PCD-CT_140keV_. All p values are corrected for multiple comparisons using Bonferroni correction. **A** entire cohort and stratified by **B** hip replacement and **C** spinal osteosynthesis. PCD-CT_Std_ = Standard reconstruction; PCD-CT_140keV_ = virtual monoenergetic reconstruction at 140 keV; PCD-CT_iMAR_ = dedicated iterative metal artifact reduction algorithm; PCD-CT_140keV+iMAR_ = dedicated iterative metal artifact reduction algorithm combined with virtual monoenergetic reconstruction at 140 keV

Repeated measure ANOVA indicated a significant impact for the type of reconstruction on artifact severity (F value = 270; df = 3; *p* < 0.001). Relative changes in HU values were as follows: PCD-CT_140keV_ = 38 ± 28.7, PCD-CT_iMAR_ = 13.5 ± 10.8 and PCD-CT_140keV+iMAR_ = 9.6 ± 7.9 with PCD-CT_Std_ = 100 ± 0.0 serving as reference. Post hoc pairwise testing showed a significant difference in artifact reduction potential between the iMAR and non-iMAR reconstructions (all *p* < 0.001), whereas no significant difference was found between PCD-CT_iMAR_ vs. PCD-CT_iMAR+140 keV_ (*p* = 0.69). A summary is presented in Fig. 5a.

Similar results were found in subgroup analysis stratified by hip replacements and spinal osteosyntheses with the only difference that PCD-CT_140keV_ facilitated a more severe artifact reduction in patients with spinal osteosyntheses compared to hip replacements (Fig. 5b and c).

## Discussion

This study explored the value of several metal artifact reduction approaches in patients with orthopedic implants using a clinically approved PCD-CT system. Our results indicate that artifacts could be significantly reduced with a dedicated iMAR algorithm as a standalone technique. Combining the iMAR approach with high keV monogenetic reconstructions at 140 keV resulted in a further improvement of image quality and diagnostic confidence compared to the clinical standard reconstruction, whereas monoenergetic images at 140 keV alone only provided satisfying results in patients with spinal osteosyntheses but not in patients with hip replacements.

This is of clinical relevance to account for impaired image quality caused by metallic implants. For orthopedic hardware, assessment of the implant itself as well as of the surrounding anatomy is clinically important to ensure the integrity of the implant and to detect potentially relevant findings in proximity that might otherwise go unnoticed [28]. In line with previous studies using conventional EID-CT systems, our results indicate that the tested iMAR algorithm alone and in combination with monoenergetic imaging at 140 keV is an effective approach to address this clinical need by providing improved image quality and diagnostic confidence compared to the standard reconstruction. This was found to be true for all evaluated subjective image criteria for iMAR as a standalone technique and could be further improved in combination with high keV monoenergetic reconstructions at 140 keV. PCT-CT_140keV_ alone did not yield significantly higher reading scores compared to the PCD-CT_Std_ in the entire cohort and can therefore not be recommended as an artifact reduction approach if iMAR is available.

However, our subgroup analysis stratified by hip replacement and spinal osteosynthesis revealed that unlike in patients with hip replacements, the 140 keV monoenergetic reconstruction facilitates a significant reduction in artifacts and improvement in image quality compared to the standard reconstruction in patients with spinal osteosynthesis. We attribute this observation to the currently missing iMAR solution optimized for spinal osteosynthesis, which makes high keV monoenergetic reconstructions a valuable alternative in those patients. These results are corroborated by a recent study on the potential of 130 keV monoenergetic reconstructions for metal artifact reduction in 32 patients with spinal osteosynthesis that also described significantly improved image quality and reduced artifacts [29].

The results of the current study complement previous investigations on metal artifact reduction using EID-CT systems. For example, Bongers et al. reported in a study including 46 patients with hip or dental implants using a second-generation dual-source CT scanner that iMAR and virtual monoenergetic reconstructions at 130 keV significantly improved subjective image quality with iMAR yielding superior results compared to the 130 keV reconstructions [15]. Another study by Han et al. including 33 patients with hip implants and 20 patients as controls found increased diagnostic confidence for the pelvic cavity in abdominopelvic CT scans with a dedicated metal artifact reduction software [30]. Similar results were found in initial studies using PCD-CT data, in which high keV monoenergetic reconstruction alone and in combination with iMAR allowed for significant artifact reduction and improved image quality in patients with hip replacement [25, 26]. In addition, several recently published studies have reported comparable findings regarding artifact reduction in patients with dental hardware [31–33]. Our results not only support these findings but also provide novel insights into artifact reduction in patients with various osteosynthesis. Firstly, we provide a systematic qualitative and quantitative analysis of different artifact reduction techniques for orthopedic implants. Secondly, our subanalysis (hip prosthesis vs. spinal implants) reveals that depending on which anatomical region is examined, the performance of the different artifact reduction techniques provide significantly different results, which has not yet been investigated for PCD-CT and needs to be considered during image reconstruction.

Reliable and effective artifact reduction techniques are also of interest for the expected adoption of novel data analysis strategies. Clinical implementation of artificial intelligence and quantitative image analysis tools is of increasing importance to support clinical workflows in handling the constantly growing amount of data acquired in daily care [34]. However, to ensure accurate and reliable post-processing results, robust input data for the analysis tools is required to avoid potentially erroneous output. Image manipulation, such as artifact reduction, poses the risk of altering the image signal/homogeneity of input data. In our CV analysis, we demonstrated that neither the iMAR approach nor the monoenergetic reconstructions significantly change the original image signal, which could be a relevant limitation for downstream post-processing tasks.

The study has the following limitations. We investigated a spectrum of different orthopedic hardware without further knowledge about vendor and material composition. Further, we included consecutive patients to investigate the artifact reduction potential of the different investigated approaches. As no implant-related findings were found in our relatively small study cohort, more focused studies are needed to assess and compare how often the artifact reduction techniques provide added value and change patient management. Further, not systematic assessment of different keV levels was performed. Finally, results of the ratio approach to quantitatively assess the artifact reduction potential need to be interpreted with caution if the HU values of the evaluated region/tissue in the corrected image approaches 0 as this may provide biased results.

In conclusion, PCD-CT allows for effective metal artifact reduction in patients with orthopedic implants resulting in superior image quality and diagnostic confidence with the potential to improve patient management and clinical decision making.

### Supplementary Information

Below is the link to the electronic supplementary material.

Supplementary file1 (DOCX 21 kb)
