# Supplementary material for: Photon-counting detector computed tomography for metal artifact reduction: a comparative study of different artifact reduction techniques in patients with orthopedic implants
Source: Radiol Med. 2024 Apr 30;129(6):890–900. doi: 10.1007/s11547-024-01822-x (PMC11168992; doi:10.1007/s11547-024-01822-x)
Supplement: Supplementary file 1 — Supplementary file1 (DOCX 21 kb) [file 11547_2024_1822_MOESM1_ESM.docx]

|  | ***PCD-CT_std_*** | ***PCD-CT_140 keV_*** | ***PCD-CT_iMAR_*** | ***PCD-CT_140keV+iMAR_*** |  |
| --- | --- | --- | --- | --- | --- |
| **Image quality** | 3 [2.67-3] | 3.3 [3-3.33] | _­_4 [3.67-4.6] | 4.3 [3.67-4.3] |  |
| ***Comparison*** | | | | | |
| PCD-CT_std_ | — |  |  |  |  |
| PCD-CT_140 keV_ | p=0.7 | — |  |  |  |
| PCD-CT_iMAR_ | P<0.001 | P<0.01 | — |  |  |
| PCD-CT_140keV+iMAR_ | P<0.001 | p=0.01 | p=1 | — |  |
| **Artifact severity** | 1.67 [1.67-2] | 2.3 [1.67-2.67] | _­_3.67 [3.3-4] | 3.67 [3.3-4] |  |
| ***Comparison*** | | | | | |
| PCD-CT_std_ | — |  |  |  |  |
| PCD-CT_140 keV_ | p=0.44 | — |  |  |  |
| PCD-CT_iMAR_ | P<0.001 | p<0.01 | — |  |  |
| PCD-CT_140keV+iMAR_ | P<0.001 | P<0.01 | p=1 | — |  |
| **Adjacent anatomy** | 1.67 [1.67-2] | 2.3 [2-2.67] | _­_3.67 [3-4] | 3.67 [3.3-4] |  |
| ***Comparison*** | | | | | |
| PCD-CT_std_ | — |  |  |  |  |
| PCD-CT_140 keV_ | p=0.13 | — |  |  |  |
| PCD-CT_iMAR_ | p<0.001 | p<0.01 | — |  |  |
| PCD-CT_140keV+iMAR_ | p<0.001 | p<0.01 | p=1 | — |  |
| **Distant anatomy** | 3 [2.6-3.3] | 4 [4-4.3] | 4 [3.67-4] | 4.3 [4-4.3] |  |
| ***Comparison*** | | | | | |
| PCD-CT_std_ | — |  |  |  |  |
| PCD-CT_140 keV_ | p<0.001 | — |  |  |  |
| PCD-CT_iMAR_ | p<0.001 | p=0.035 | — |  |  |
| PCD-CT_140keV+iMAR_ | p<0.001 | p=1 | p=0.038 | — |  |
| **Diagnostic confidence** | 1.6 [1.6-2] | 2.6 [2-3] | _­_3.6 [3.3-4.3] | 3.6 [3.6-4] |  |
| ***Comparison*** | | | | | |
| PCD-CT_std_ | — |  |  |  |  |
| PCD-CT_140 keV_ | p=0.17 | — |  |  |  |
| PCD-CT_iMAR_ | p<0.001 | p<0.01 | — |  |  |
| PCD-CT_140keV+iMAR_ | p<0.001 | p=0.02 | p=1 | — |  |

**Table S1.** Results of the qualitative image analysis in stratified analysis by hip replacement

PCD-CT_Std_=Standard reconstruction; PCD-CT_140keV_=virtual monoenergetic reconstruction at 140keV; PCD-CT_iMAR_= dedicated iterative metal artifact reduction algorithm; PCD-CT_140keV+iMAR_= dedicated iterative metal artifact reduction algorithm combined with virtual monoenergetic reconstruction at 140keV

|  | ***PCD-CT_std_*** | ***PCD-CT_140 keV_*** | ***PCD-CT_iMAR_*** | ***PCD-CT_140keV+iMAR_*** |  |  |
| --- | --- | --- | --- | --- | --- | --- |
| **Image quality** | 3 [2.75-3.3] | 3.67 [3.4-3.92] | _­_4 [3.3-4] | 4.5 [3.75-4.67] | |  |
| ***Comparison*** | | | | | | |
| PCD-CT_std_ | — |  |  |  | |  |
| PCD-CT_140 keV_ | p=0.03 | — |  |  | |  |
| PCD-CT_iMAR_ | p=0.02 | p=1 | — |  | |  |
| PCD-CT_140keV+iMAR_ | p<0.01 | p=0.3 | p=0.53 | — | |  |
| **Artifact severity** | 2.3 [1.67-2] | 3.3 [1.67-2.67] | _­_3.5 [3.3-4] | 4.3 [3.3-4] | |  |
| ***Comparison*** | | | | | | |
| PCD-CT_std_ | — |  |  |  | |  |
| PCD-CT_140 keV_ | p<0.01 | — |  |  | |  |
| PCD-CT_iMAR_ | p<0.01 | p=1 | — |  | |  |
| PCD-CT_140keV+iMAR_ | p<0.001 | p=0.12 | p=0.17 | — | |  |
| **Adjacent anatomy** | 2.3 [2-2.92] | 3.33 [3-4] | _­_3.5 [2.75-4] | 4.3 [3.42-4.58] | |  |
| ***Comparison*** | | | | | | |
| PCD-CT_std_ | — |  |  |  | |  |
| PCD-CT_140 keV_ | p<0.01 | — |  |  | |  |
| PCD-CT_iMAR_ | p<0.01 | p=1 | — |  | |  |
| PCD-CT_140keV+iMAR_ | p<0.001 | p=0.38 | p=0.57 | — | |  |
| **Distant anatomy** | 3 [2.67-3.3] | 4 [4-4.58] | 4.17 [3.67-4.3] | 4.17 [3.42-4.3] | |  |
| ***Comparison*** | | | | | | |
| PCD-CT_std_ | — |  |  |  | |  |
| PCD-CT_140 keV_ | p<0.001 | — |  |  | |  |
| PCD-CT_iMAR_ | p<0.001 | p=1 | — |  | |  |
| PCD-CT_140keV+iMAR_ | p<0.001 | p=1 | p=1 | — | |  |
| **Diagnostic confidence** | 2.5 [2.08-2.92] | 3.67 [3.33-4] | ­4 [3-4.25] | 4.3 [3.75-4.67] | |  |
| ***Comparison*** | | | | | | |
| PCD-CT_std_ | — |  |  |  | |  |
| PCD-CT_140 keV_ | p<0.01 | — |  |  | |  |
| PCD-CT_iMAR_ | p<0.01 | p=1 | — |  | |  |
| PCD-CT_140keV+iMAR_ | p<0.01 | p=0.72 | p=1 | — | |  |

**Table S2.** Results of the qualitative image analysis in stratified analysis by spine instrumentation

PCD-CT_Std_=Standard reconstruction; PCD-CT_140keV_=virtual monoenergetic reconstruction at 140keV; PCD-CT_iMAR_= dedicated iterative metal artifact reduction algorithm; PCD-CT_140keV+iMAR_= dedicated iterative metal artifact reduction algorithm combined with virtual monoenergetic reconstruction at 140keV
